# Experiment and analysis of physical, mechanical, and viscoelastic properties of the roots and stalks of green leafy vegetables

**DOI:** 10.1371/journal.pone.0305572

**Published:** 2024-07-02

**Authors:** Yue Jin, Zhiyu Song, Renlong Zhang, Jianfei Zhang

**Affiliations:** Ministry of Agricultural and Rural Affairs, Nanjing Institute of Agricultural Mechanization, Nanjing, China; Industrial University of Ho Chi Minh City, VIET NAM

## Abstract

Green leafy vegetables are an essential component of Chinese leafy vegetables. Due to their crisp stems and tender leaves, orderly harvester generally causes significant mechanical clamping damage. The physical and mechanical properties of green leafy vegetables are one of the important basis to design the orderly harvester. At the same time, they provide important parameters for the simulation and optimization of harvester. So, this paper measured the physical characteristic parameters of roots and stems of green leafy vegetables. Then, based on the TMS-Pro texture analyzer, the elasticity modulus of the roots and stems of green leafy vegetables were measured. The static friction coefficient, dynamic friction coefficient, and restitution coefficient of green leafy vegetables root-root, stem-stem, root-steel, and stem-steel were measured separately using a combination method of inclined plane and high-speed photography. Uniaxial compression creep experiments were carried out on whole and single leaf of green leafy vegetables using the TA.XT plus C universal testing machine. The constitutive equation of the four-element Burgers model was used to fit the deformation curve of the sample with time during the constant-pressure loading stage. The fitting determination coefficients R^2^ were all higher than 0.996, which verified the reasonable validity of the selected model. The above experimental results provide a parameter basis and theoretical support for the design and discrete element simulation optimization of orderly harvester critical components of green leafy vegetables.

## 1. Introduction

Green leafy vegetables are fast-growing vegetables that mainly produce fresh and tender green leaves, petioles, and stems. Because of their short growth period, fresh and delicate taste, and rich nutrition, they are widely planted throughout the four seasons in various parts of China. Consequently, these vegetables are essential components of vegetables. In recent years, with the adjustment of the rural industrial structure and the rapid development of the vegetable industry, the requirements of people for the freshness, safety quality, taste, and flavor of green leafy vegetables have generally increased, and the demand for green leafy vegetables has also sharply increased [1, 2].

The scientific name for green leafy vegetables is Brassica campestris L.ssp.chinensis Makino. The most familiar green leafy vegetables on the market are represented by the cruciferous species Shanghai Qing. The stems and leaves of these species are usually wrapped in 3–4 layers from the outside to the inside, with each layer consisting of four stems and leaves distributed staggered between layers. Its roots mainly comprise the primary root and the fibril root system, with a higher straightness of the primary root. Usually, the root diameter at the junction of the root and stem is the thickest, and the end is the thinnest, forming an irregular cone. The fibril root system is a network interwoven between roots and between roots and soil, creating a complex multiphase composite structure.

In mechanized and orderly harvesting, contact between the stems and leaves of green leafy vegetables and mechanical components is inevitable. This can easily cause mechanical damage, directly leading to decreased yield, quality, and equipment price harvested from green leafy vegetables. The main factor restricts the development of comprehensive mechanized production of green leafy vegetables throughout the entire [3–5]. Liu and Li et al. carried out compression and shear mechanics experiments on the stalks of Chinese little greens and the roots of spinach respectively with the aid of a universal testing machine [5, 6]. Zou et al. conducted a compression creep experiment on the whole spinach by using the texture analyzer [7]. At present, the physical and mechanical properties of green leafy vegetables such as Shanghai Qing are not studied systematically and deeply. Therefore, this article conducts in-depth research on the physical and mechanical properties of green leafy vegetables, which can provide necessary theoretical and parameter support for designing and optimizing critical devices of orderly harvesters. Improving the quality of mechanized and orderly harvesting operations and reducing mechanical damage are crucial.

## 2. Study of the physical characteristics of green leafy vegetables

**Fig 1:**
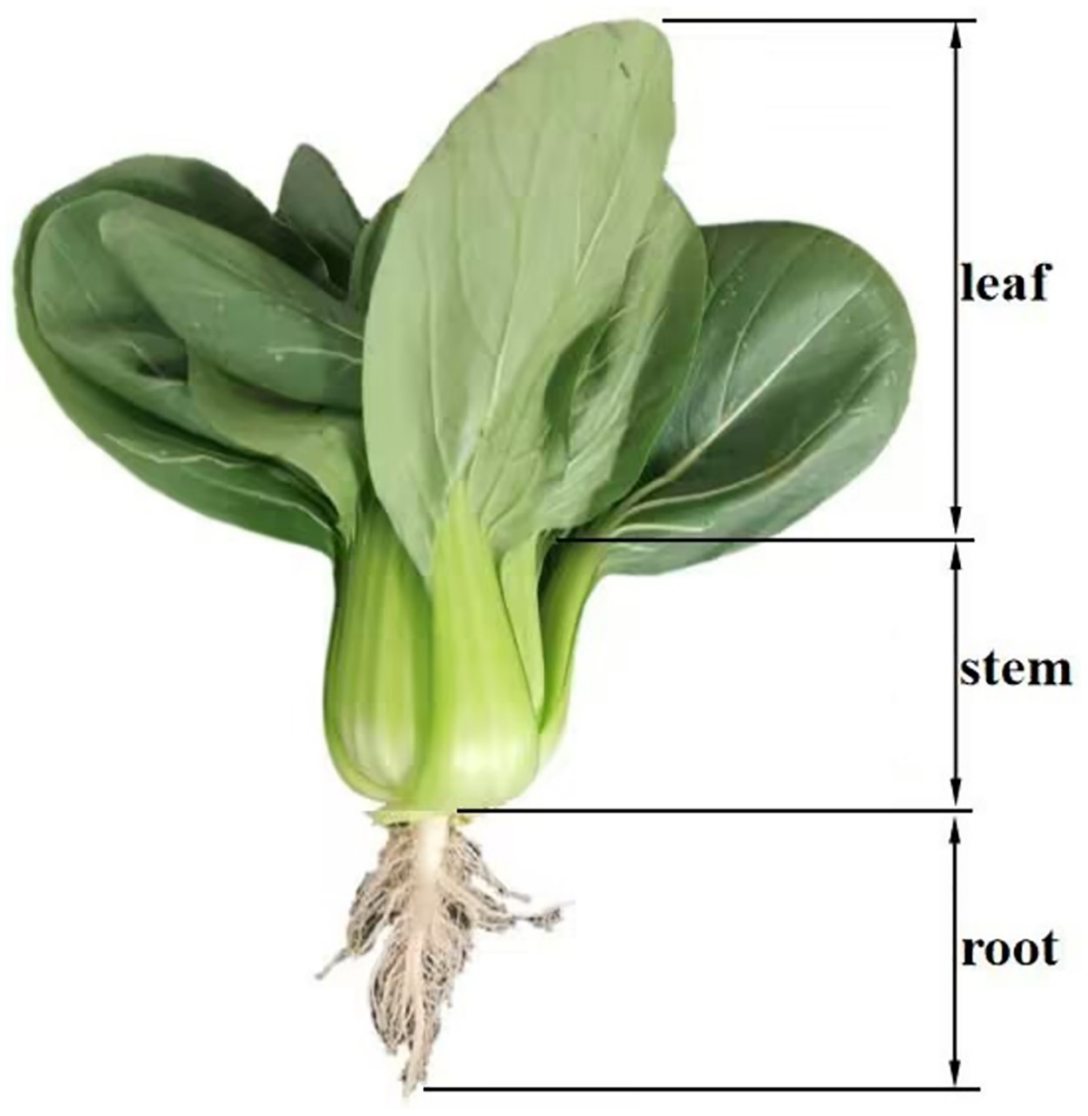
Photos of green leafy vegetable samples.

Studying the physical and mechanical properties of green leafy vegetables is a necessary theoretical and design bases for developing harvesters because green leafy vegetables are the direct target of orderly harvesters. In April 2023, the physical and mechanical properties of green leafy vegetables samples were tested in the laboratory of Nanjing Institute of Agricultural Mechanization, Ministry of Agricultural and Rural Affairs. The experimental variety of green leafy vegetables was Shanghai Qing Zhenpin 66. Ten samples of green leafy vegetables with relatively consistent growth were selected (Fig 1). Instruments, such as a vernier caliper (with an accuracy of 0.02 mm), a balance (with an accuracy of 0.0001 g), and a measuring cylinder (with an accuracy of 1 ml), were used to measure and statistically analyze the primary physical, morphological parameters of green leafy vegetables, including stem and leaf length, stem diameter, root length, maximum root diameter, stem and leaf density, and root density. The average value of each sample was calculated after five repeated measurements. After measurement, the length and weight of the stems and leaves of green leafy vegetables are significantly different from their roots (Table 1). Studying the physical characteristics of green leafy vegetables can provide a theoretical basis for developing, improving, and optimizing ordered harvesters.

**Table 1:** Physical characteristics parameters of green leafy vegetables.

Variety	Stem leaf			Root			
Zhenpin 66	Length mm	Mass g	Density kg·m^-3^	Maximum diameter mm	Length mm	Mass g	Density kg·m^-3^
**Minimum value**	160	72.9	760	7.86	80.8	2.04	1100
**Maximum value**	185	117.8	847	10.8	110.44	2.75	1275
**Average value**	176.53	89.97	806.20	8.88	97.39	2.44	1174.80
**Standard deviation**	11.62	15.51	33.32	1.24	12.95	0.28	80.72
**Variation coefficient**	0.07	0.17	0.04	0.14	0.13	0.11	0.07

## 3. Study on the mechanical properties of green leafy vegetables

### 3.1 Experiment scheme

#### 3.1.1 Compression experiment

**Fig 2:**
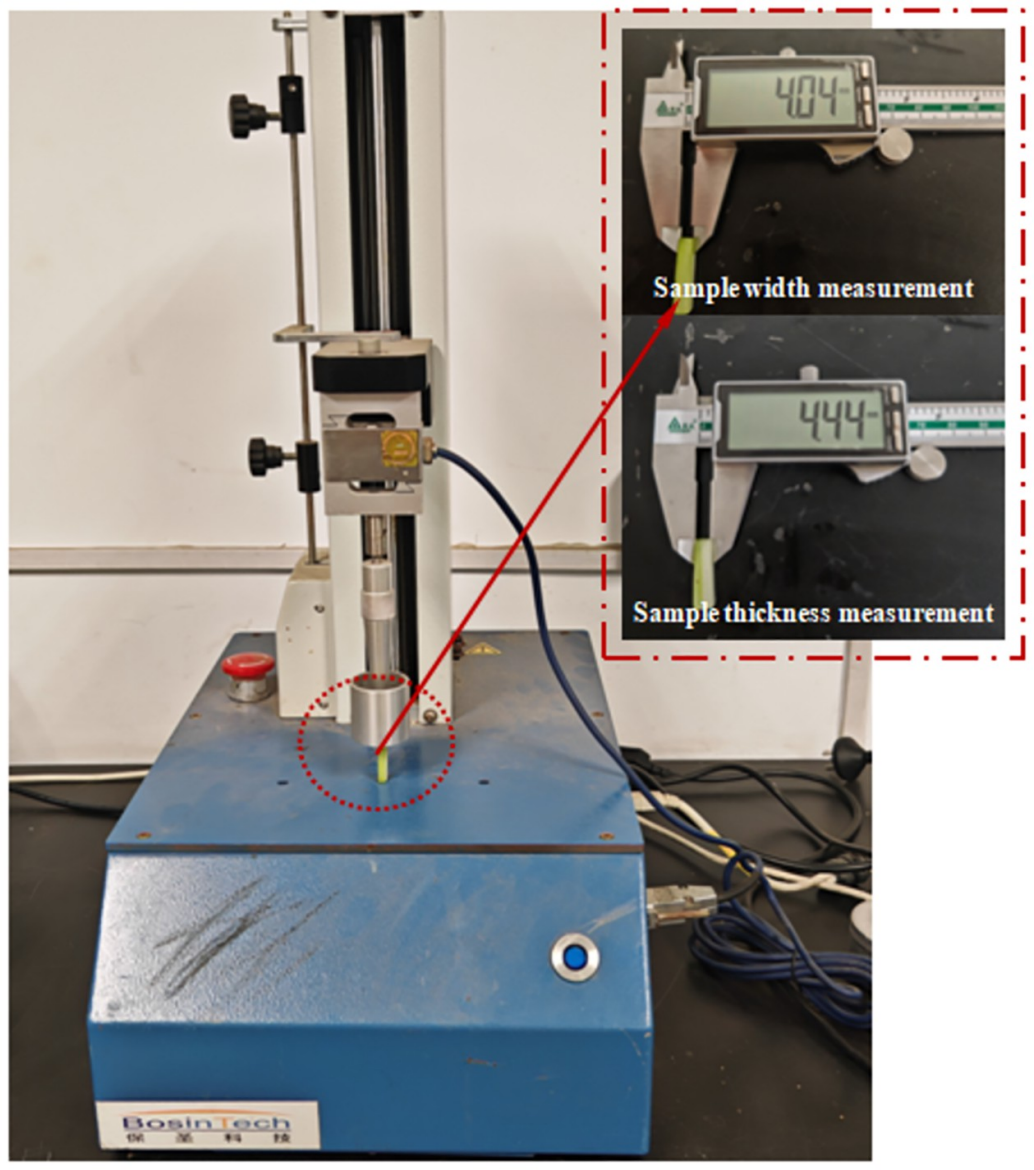
Stem sample compression experiment of green leafy vegetable.

The elastic properties of plants are the same in any plane in the axial direction because they are mostly transversely isotropic materials but different in two directions perpendicular to each other in the axial and radial directions [7, 8]. This article conducted axial compression experiments on the roots and stems of green leafy vegetables. The roots and stems of green leafy vegetables Zhenpin 66 were preprocessed to avoid the significant impact of irregular shapes on the experimental results. Based on the principle of retaining regular radial diameter segments, the root samples were cut into 7.5±1.0mm×6.5±1.0mm (diameter × length) cylinder, the stem samples were cut into 4.0±0.5mm×4.5±0.5mm (width ×thickness) rectangular narrow strip. Compression characteristics experiments were conducted on a TMS-Pro texture analyzer (Fig 2).

Before the experiment started, 30 root and stem samples were selected, the original diameter and length of the root samples and the original length and width of the stem samples were measured. The texture analyzer was activated, the initial height was calibrated, the force and displacement measurement was zeroed. The sample was placed on a texture analyzer and equipped with a disc-shaped particular pressure head (diameter 100mm) for compression. The compression rate of the pressure head was set to 0.1 mm/s, and the data collection frequency was 100 pps. When the sample was compressed to rupture, the experiment ended.

#### 3.1.2 Static and dynamic friction coefficient measurement experiment

**Fig 3:**
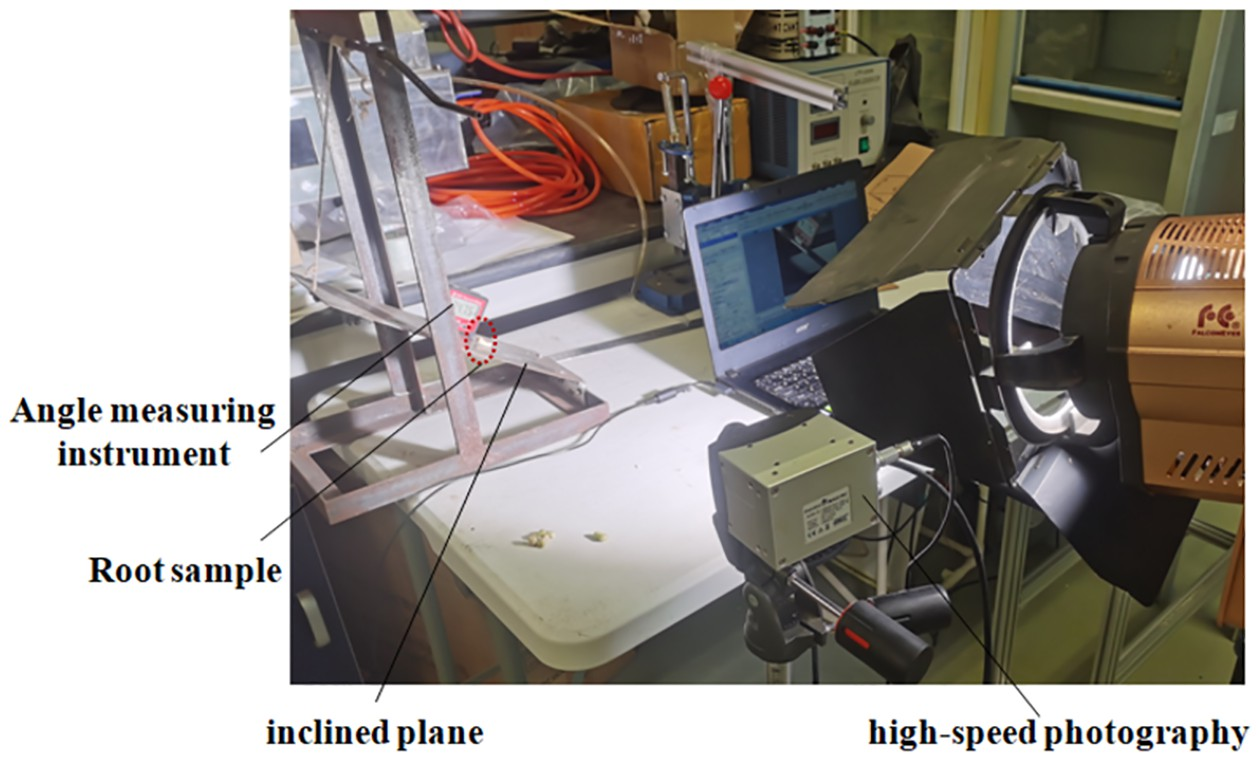
Friction coefficient measurement.

The static and dynamic friction coefficients between the roots and stems of green leafy vegetables and the test materials were measured using the slope method combined with high-speed photography (Fig 3). First, the samples of green leafy vegetables Zhenpin 66 was placed on the bevel of the contact material, and the angle measuring instrument was placed at the side of the bevel. Then the bevel was raised slowly, the angle between the contact material and the horizontal plane was increased gradually. When the sample began to slide down or roll on the bevel of the contact material, the bevel was stopped lifting. The bevel angle, rolling distance and time under corresponding circumstances were found out by replaying the collected images of the high-speed photography. The calculation was conducted according to formulas (1) and (2) [9–11].

μ=tanθ (1)

where *μ*—Static friction coefficient between the sample of green leafy vegetables and inclined plane materials. *θ*—The inclination angle of the inclined plane material wall when the sample moves from rest (°), which is the static friction angle of the sample.

$$\mu_{k}=\text{tan}\;\alpha -\frac{a}{g\;\text{cos}\;\alpha }$$ (2)

where *μ*_*k*_—dynamic friction coefficient between the sample of green leafy vegetables and inclined plane materials. *a*—acceleration of the sample, m/s^2^. *α*—The inclination angle of the inclined plane material wall when the sample moves from rest to rolling (°).

#### 3.1.3 Collision experiment

**Fig 4:**
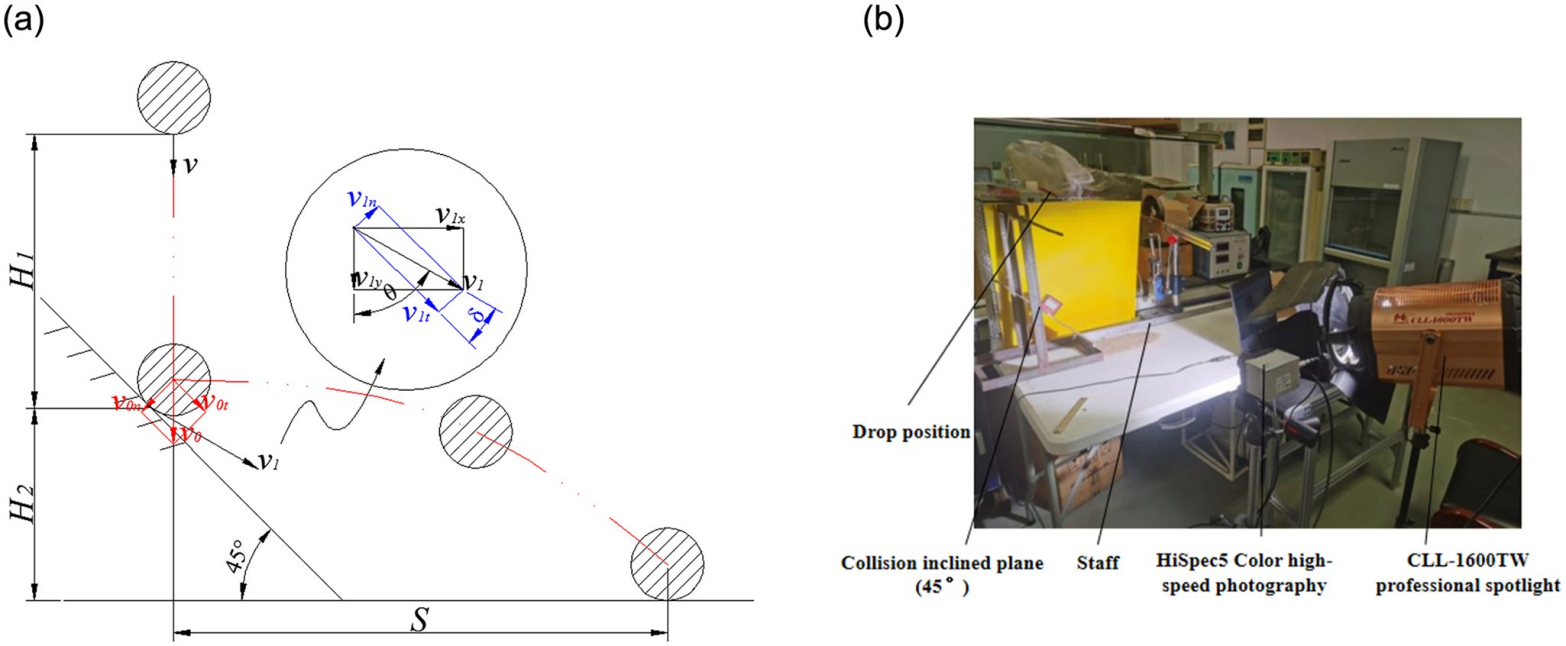
Restitution coefficient measurement.

The collision experiments of roots and stems of green leafy vegetables Zhenpin 66 were conducted by combining inclined plane collision and high-speed photography (Fig 4). The collision experiments were able to measure the restitution coefficients between the samples and the different collision materials [7, 12, 13]. For instance, the restitution coefficient between roots and inclined plane materials was investigated using the most common collision velocity ratio to calculate the restitution coefficient of the roots. The measurements were made to calculate the normal relative approach velocity and the normal relative separation velocity of the root at the point of contact before and after the collision [14], as shown in Eq 3.

$$e=\frac{v_{1n}}{v_{0n}}=\frac{v_{1}\times \text{sin}\;\delta }{v_{0}\times \text{sin}\;45^{{}^{\circ}}}$$ (3)

where *e*—coefficient of recovery for collision between green leafy vegetable root and inclined plane material. *v*_1*n*_—Normal relative separation velocity of the contact point after collision, m/s. *v*_0*n*_—normal relative approach velocity of the contact point before the collision, m/s.

Since the root is in a free-fall motion (ignoring the effect of air resistance) before the collision with the inclined plane, the instantaneous velocity of the root before the collision was at the moment of contact with the inclined plane:

$$v_{0}=\sqrt{2gH_{1}}$$ (4)

where *H*_1_—root drop height, mm.

When the root collided with the inclined plane, it was ejected along the plane for a horizontal distance of length S when the instantaneous velocity after the collision was:

$$v_{1}=\sqrt{v_{1x}^{2}+v_{1y}^{2}}$$ (5)

where *v*_1*x*_—fractional velocity in the x-direction after root collision, m/s. *v*_1*y*_—Fractional velocity in the y-direction after root collision.

Included among these:

$$\left\{\begin{array}{c} v_{1x}=\frac{S}{t}\\ v_{1y}=\frac{\left(H_{2}-\frac{1}{2}gt^{2}\right)}{t} \end{array}\right.$$ (6)

where *S*—distance along the horizontal direction of ejection after root collision, mm. *H*_2_—root fall height after collision, mm. *t*—root post collision motion time (s).

Substituting Eqs 4–6 into Eq 3 yielded the coefficient of recovery for collision between the root of green leafy vegetables and the inclined material, viz.:

$$e=\frac{\sqrt{{\text{S}}^{2}+{\left({\text{H}}_{2}-\frac{1}{2}{\text{gt}}^{2}\right)}^{2}}\times \text{sin}\;\delta }{\sqrt{2gH_{1}}\times \text{sin}\;45^{{}^{\circ}}\times \text{t}}$$ (7)

where *δ*—Angle between the contact point velocity and its tangential partial velocity after collision (°).

In order to ensure the accuracy of the measurement results of static friction coefficient, dynamic friction coefficient and collision recovery coefficient, 30 root and stem samples of green leafy vegetables were selected and repeated three times to take the average value to arrive at the final measurement results.

### 3.2 Experimental results analysis

#### 3.2.1 Compression experiment results analysis

**Fig 5:**
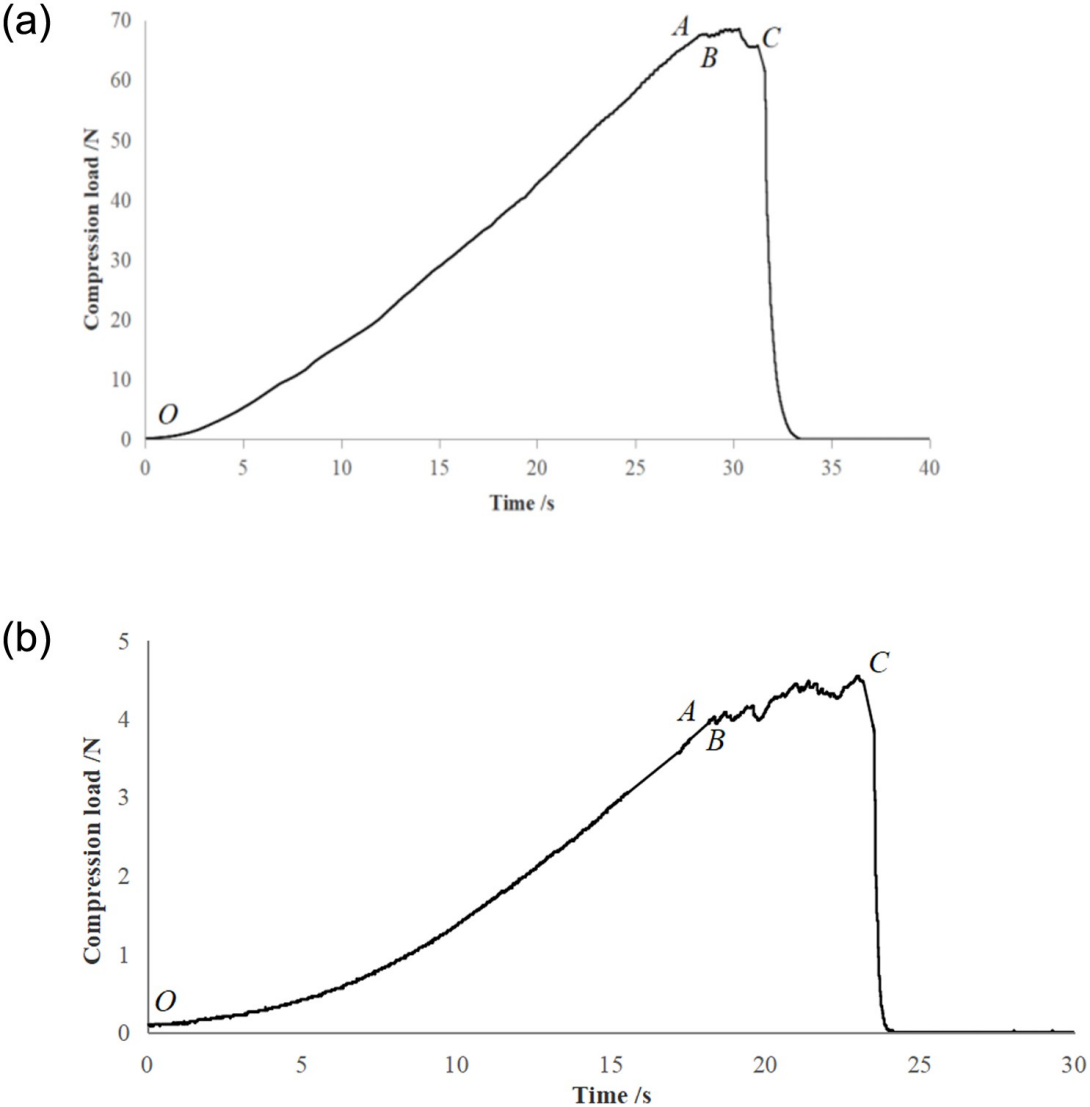
Compression load-time curve. *a*. Axial compression of the roots of green leafy vegetables. *b*. Axial compression of the stems of green leafy vegetables.

Due to length limitations, this study only lists the compression test results of the roots and stems of green leafy vegetables with sample number 1. The curve of the axial load of the roots and stems of green leafy vegetables over time is shown in Fig 5. The elastic modulus of the roots and stems of green leafy vegetables can be obtained through axial compression experiments. The *OA* section is the stage when the texture analyzer starts to compress the sample. At this stage, the sample presents linear elastic deformation. The slope of the straight line section is found out, and the compressive elastic modulus *E* can be calculated from the following equation [15, 16]:

$$E=\frac{F}{A\varepsilon }$$ (8)

where *E* is the elastic modulus of the sample, Pa. *F* is the pressure, N. *A* is the original compressed area, m^2^. *ε* is the linear strain.

The axial compression process was divided into three stages (Fig 5): the first stage was the elastic stage, with the pressure unchanged. The second stage was the linear elastic stage, where the pressure increased with time and displacement and changed linearly. The third stage was the failure stage. When the pressure reached the limit of the compressive ratio, the sample was destroyed, and the load suddenly dropped, leading to mechanical damage. The compressive limit that the roots of green leafy vegetables withstand was much greater than the stems, comparing the two curves in Fig 2*a* and 2*b*. This indicated that the stems are more prone to mechanical damage. The elastic modulus of the roots of green leafy vegetables was calculated using formula (8) as 6.02 × 10^5^ Pa, and the elastic modulus of the stem was 9.16 × 10^5^ Pa.

#### 3.2.2 Friction coefficient and collision recovery coefficient experiment results analysis

The measured results of the static friction coefficient, dynamic friction coefficient, and restitution coefficient of green leafy vegetable root-to-root, stem-to-stem, root-to-steel plate, and stem-to-steel plate are shown in Table 2. The restitution coefficients between green leafy vegetable stems were observed in the measurement results. Test materials were smaller than those between roots and the same test materials. This indicated that when green leafy vegetable stems and test materials collided, the stem deformation recovery ability was small, and the stems were more prone to damage. The experimentally measured static friction coefficients of the maximum value were the stems of green leafy vegetables with stems, and the minimum value was those of the steel plates of green leafy vegetables. The maximum value of the dynamic friction coefficients was the stems of green leafy vegetables. The minimum value was between the root and the root of the green leafy vegetables. This result provided data support for improving discrete element modeling and optimizing the orderly harvesting equipment of green leafy vegetables.

**Table 2:** Measured correlation coefficients of roots and stems of green leafy vegetables.

Category	Static friction coefficient				Dynamic friction coefficient				Restitution coefficient			
	Root-root	Stem-stem	Root-steel	Stem-steel	Root-root	Stem-stem	Root-steel	Stem-steel	Root-root	Stem-stem	Root-steel	Stem-steel
**Minimum value**	0.67	1.10	0.81	0.37	0.12	0.40	0.29	0.22	0.36	0.31	0.48	0.45
**Maximum value**	1.23	1.33	0.99	0.78	0.36	0.75	0.43	0.45	0.61	0.56	0.76	0.68
**Average value**	1.07	1.25	0.89	0.65	0.24	0.54	0.36	0.32	0.52	0.43	0.61	0.53
**Standard deviation**	0.2	0.15	0.08	0.07	0.1	0.13	0.05	0.09	0.12	0.08	0.1	0.08

## 4. Studies on the viscoelastic properties of green leafy vegetables

Most fruits and vegetables, including green leafy vegetables, are essentially viscoelastic bodies with both elastic and viscous deformations of two mechanisms. In these mechanisms [17–21], elastic deformation refers to stems and leaves that can be completely recovered after the withdrawal of the applied external force. The viscous deformation refers to the deformation part of the stem and leaves that cannot be recovered and retained after the withdrawal of the applied external force, also called plastic deformation. When green leafy vegetables are mechanized and harvested orderly, the mechanical nature of the plastic deformation of the stem and leaf, and the mechanical damage, is the amplitude of the stress generated. This occurs because the extrusion of the clamping mechanism exceeds the compressive limit of the stem and leaf. Therefore, the stem and leaf cannot completely recover from deformation after leaving the clamping mechanism, generating damage.

**Fig 6:**
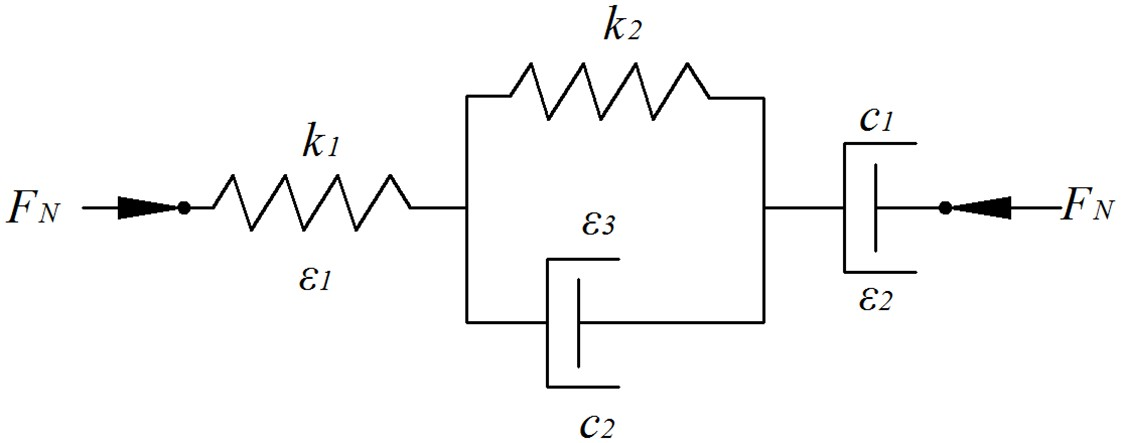
Four-element Burgers model.

Relevant literature shows that constructing a rheological mathematical model to describe the viscoelastic properties of green leafy vegetables is conducive to analyzing the deformation characteristics of green leafy vegetables under loading conditions. The four-element Burgers model is recognized as a more accurate and complete mathematical model used to characterize the viscoelastic properties of green leafy vegetables [21–23], as shown in Fig 6, and its eigenstructure equations are as follows:

$$D(t)=\frac{F_{0}}{k_{1}}+\frac{F_{0}}{c_{1}}t+\frac{F_{0}}{k_{2}}\left(1-e^{-\frac{k_{2}}{c_{2}}t}\right)$$ (9)

where *D(t)*—deformation, mm; t-time, s, *F*_*0*_—constant load, N, *k*_*1*_—instantaneous elasticity coefficient, N/mm, *k*_*2*_—delayed elasticity coefficient, N/mm, *c*_*1*_—tandem coefficient of viscosity, N·s/mm, and *c*_*2*_—parallel coefficient of viscosity, N·s/mm.

### 4.1 Creep experiment method

The creep experiment is a standard test to obtain the viscoelastic parameters of green leafy vegetables. Five whole and single leaf of Zhenpin 66 green leafy vegetables were randomly selected and placed horizontally across the indenter and base of the universal testing machine. Conveyor belts were wound above both the indenter and the base to simulate the real force of the green leafy vegetables in the clamping device. The experiments were conducted one by one. The experimental apparatus included a TA.XT plusC universal testing machine from SMS, a P/1S spherical indenter (1 in ball diameter), an industrial computer, and vernier calipers.

**Fig 7:**
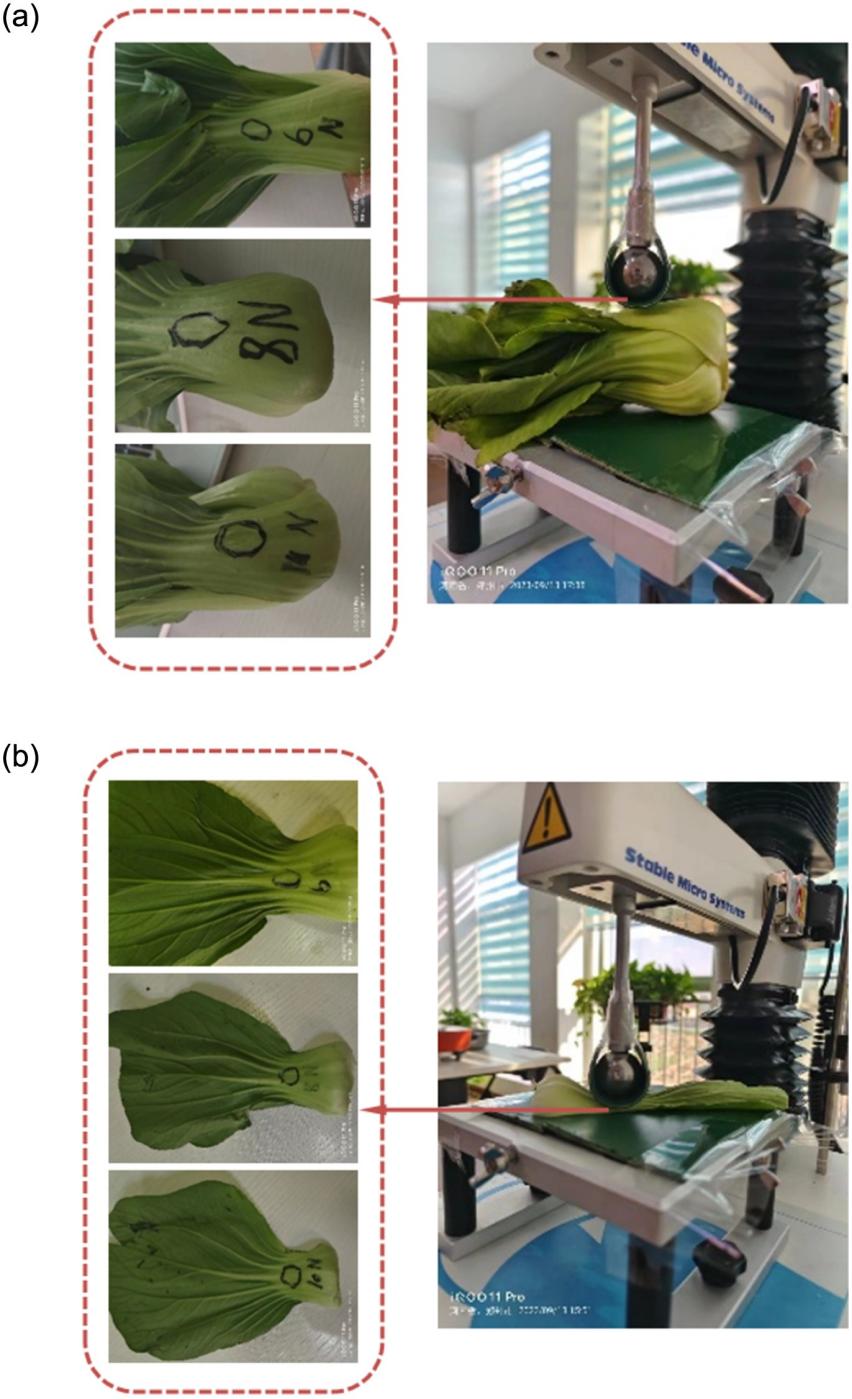
The creep experimental test. *a*. whole green leafy vegetable experiment *b*. single leaf experiment.

Constant pressure loading creep experiments were conducted to avoid the overshoot phenomenon when the loading process reached the set pressure for the first time. The indenter loading speed was set at 5 mm/min, and the data acquisition frequency was 200 pps. Three sample points were randomly selected for each specimen. The constant loading pressures of 6 N, 8 N, and 10 N were set for the three sample points to conduct the uniaxial compression creep test (Fig 7). The constant pressure loading time was 60 s.

### 4.2 Test results analysis

Creep experiments can obtain the relationship curve of the deformation of green leafy vegetables with time under constant pressure using the cftool curve fitting toolbox in the MATLAB data analysis software [24, 25]. The curve fitting of the creep experimental data of green leafy vegetables was conducted through the fitting function y = a × (1 − *e*^*bx*^) + *cx* + *d*. Four unknown parameters were also used as the constraint fitting conditions during the fitting process to improve the accuracy of the curve fitting results. The start point, lower, and upper parameters were adjusted according to each fitting result until more accurate fitting results were obtained. The four unknown parameters were also used as constraints in the fitting process to improve the accuracy of the curve-fitting results.

**Fig 8:**
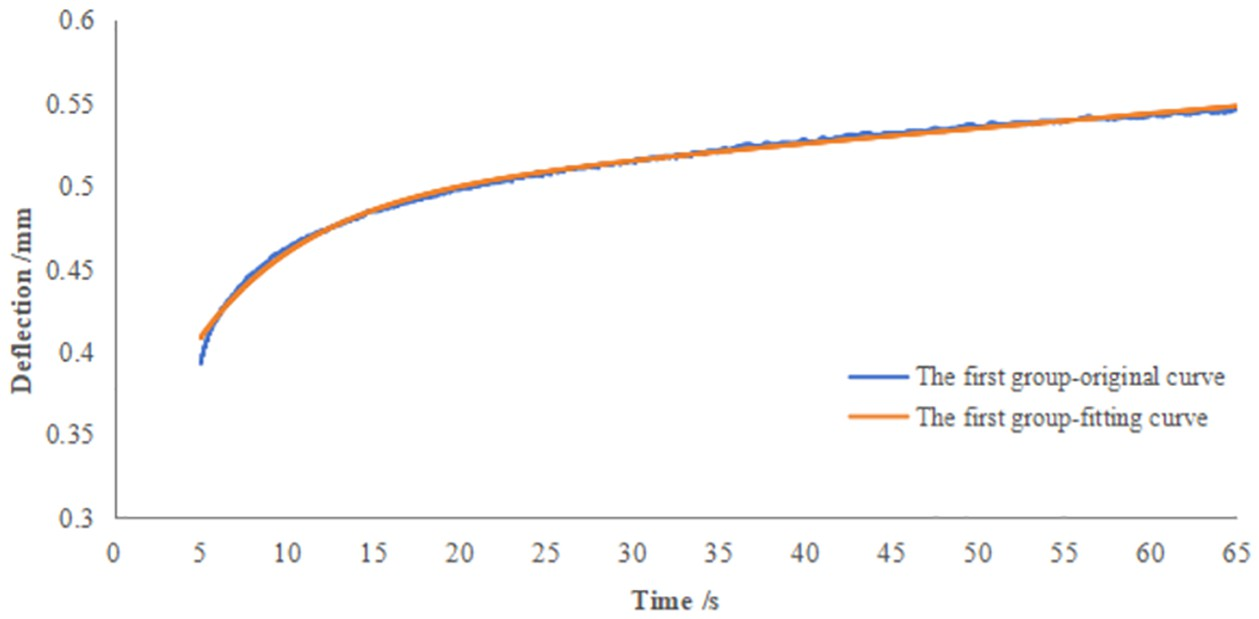
Comparison of fitting results of sample deformation versus time curves.

The start point, lower, and upper parameters were adjusted according to the fitting results each time until more accurate, appropriate results were obtained. Due to the limitation of the length of the study, this manuscript only listed the experimental fitting results of creep of the whole and single leafy of green leafy vegetables with a sample number of 1, as shown in Fig 8. It derived the values of the four unknown parameters in the fitting function and converted the parameters according to the mathematical model of viscoelastic rheology (Eq 9) to obtain the specific values of *k*_*1*_, *k*_*2*_, *c*_*1*,_*c*_*2*_.

Tables 3 and 4 show the results of parameter fitting for the creep experiments of whole and single green leafy vegetables, respectively. The intrinsic equations for their rheological properties were $D(t)=\frac{F_{0}}{19.05}+\frac{F_{0}}{2348.04}t+\frac{F_{0}}{7.44}\left(1-e^{-0.098t}\right)$ and $D(t)=\frac{F_{0}}{23.59}+\frac{F_{0}}{8418.07}t+\frac{F_{0}}{36.49}\left(1-e^{-0.15t}\right)$. From the relationship curve fitting results, the coefficients of determination *R*^*2*^ were higher than 0.996, which verified the reasonable validity of the selected model.

**Table 3:** Viscoelastic correlation coefficients of whole green leafy vegetables.

Loading force *F*_*0*_	*k*_*1*_/ (N·mm^-1^)	*k*_*2*_/ (N·mm^-1^)	*c*_*1*_/ (N·s·mm^-1^)	*c*_*2*_/ (N·s·mm^-1^)	SSE	*R* ^2^
**6N**	12.72	9.80	1982.45	95.86	0.0879	0.9991
**8N**	17.58	7.19	2394.56	74.31	0.0880	0.9993
**10N**	26.86	5.34	2667.12	57.00	0.0892	0.9994
**Average value**	19.05	7.44	2348.04	75.72	0.0884	0.9993

**Table 4:** Viscoelastic correlation coefficients of single green leafy vegetables.

Loading force *F*_*0*_	*k*_*1*_/ (N·mm^-1^)	*k*_*2*_/ (N·mm^-1^)	*c*_*1*_/ (N·s·mm^-1^)	*c*_*2*_/ (N·s·mm^-1^)	SSE	*R* ^2^
**6N**	24.12	39.44	6757.17	256.12	0.0325	0.9968
**8N**	23.48	40.15	8223.36	271.45	0.0332	0.9975
**10N**	23.16	29.87	10273.69	201.52	0.0316	0.9977
**Average value**	23.59	36.49	8418.07	243.03	0.0324	0.9973

Fig 8 shows the relationship curve between the deformation of whole and single leaves of green leafy vegetables with time under constant pressure. Table 4 shows the calculated values of the deformation of green leafy vegetables under constant pressure for 60 s. From the graphic curves and data, the initial displacement when reaching the set loading pressure varied significantly for each sample tested because the thickness of single-leaf was different in the single-leaf test, and the gaps of between each leaf was different in the whole green leafy vegetables test. These differences led to a significant difference in the initial displacement.

**Fig 9:**
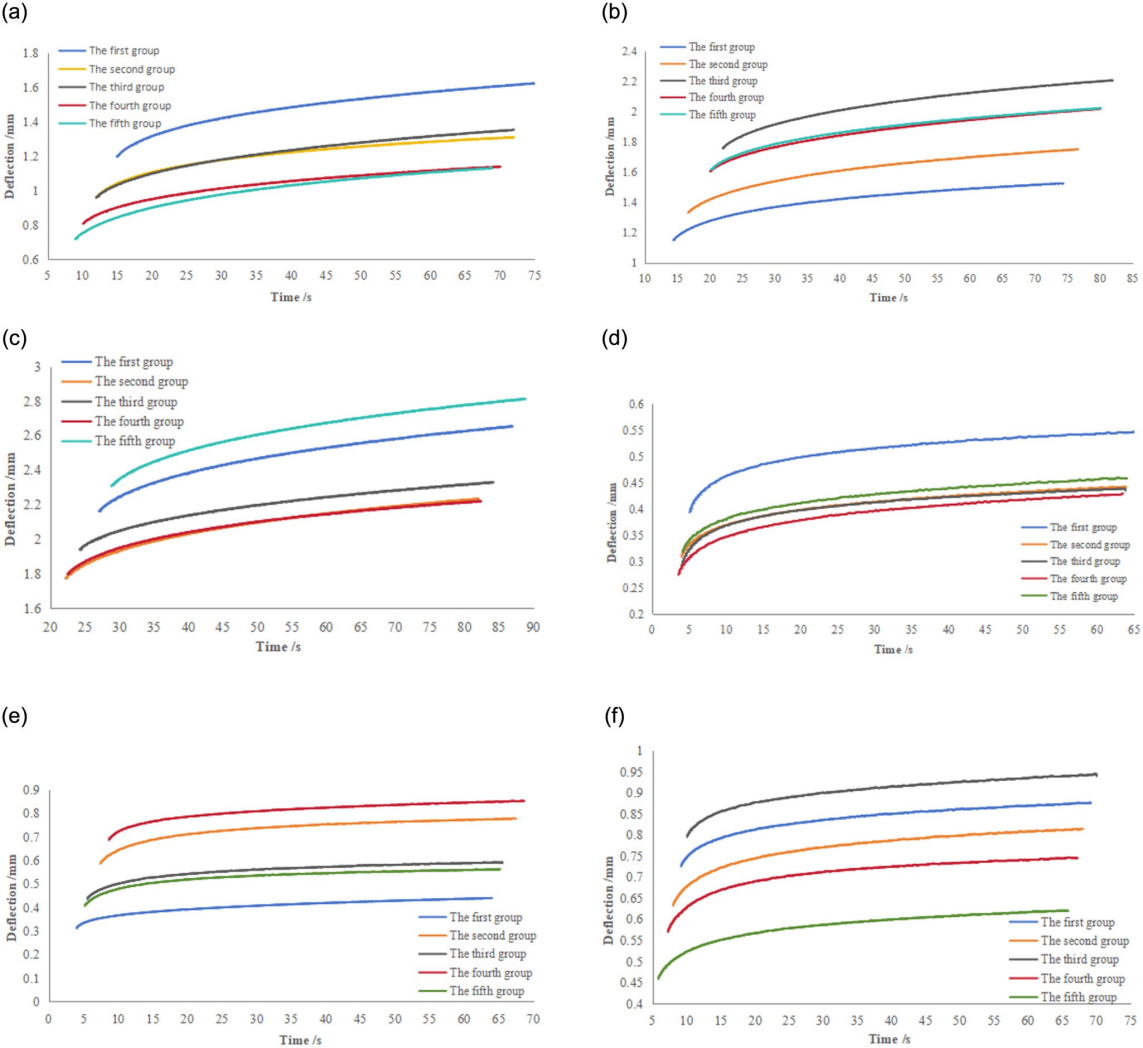
The deformation curve of green leafy vegetables under constant pressure as a function of time. *a*. Loading constant pressure 6 N (whole leaves).*b*. Loading constant pressure 8 N (whole leaves). *c*. Loading constant pressure 10 N (whole leaves).*d*. Loading constant pressure 6 N (single leaf). *e*. Loading constant pressure 8 N (single leaf). *f*. Loading constant pressure 10 N (single leaf).

The damage to green leafy vegetables under different pressures was also different (Fig 9). The slopes of the deformation versus time curves of similar samples under the same loading constant pressure were the same. This result indicates that the difference in the initial displacement did not affect the deformation from the point of view of the total deformation. From Table 5, the larger the value of the loading constant pressure set for the creep experiment, the larger the total deformation of the sample under the same loading time. When the loading constant pressure was less than 8 N, due to the large gap inside the green leafy vegetables, the deformation amount was larger than that of a single leaf when the loading force was small. When the loading constant pressure was 10 N, the whole green leafy vegetables needed to be further pressed, and the gap narrowed to reach the experimental set pressure.

**Table 5:** Deformation of green leafy vegetables under constant pressure.

Item	Whole leaves			Single leaf		
	6N	8N	10N	6N	8N	10N
**maximum value**	0.424	0.448	0.504	0.161	0.436	0.545
**minimum value**	0.330	0.372	0.389	0.128	0.264	0.422
**average value**	0.381	0.410	0.453	0.148	0.353	0.505
**Standard deviation**	0.041	0.027	0.048	0.014	0.069	0.050
**coefficient of variation**	10.656	6.674	10.645	9.380	19.572	9.867

At this time, the creep experiments showed that the deformation amount of the whole sample was smaller than that of a single leaf in the sample under constant pressure. The deformation occurred with the extension of the loading action time and nonlinear increase, and the curve was satisfied with the Burgers model of the ontological equation relationship.

## 5. Discussion

Research on the physical properties of green leafy vegetables and their roots mainly comprises primary and fibril root systems. The main root straightness was high. Usually, the root diameter of the combined root and stem part of the root is the thickest, the end of the thinnest, into an irregular cone. The fibril root system is reticulated, and the roots are interwoven among each other, the root system, and the soil to form a complex multiphase composite structure. The reticulate fibril root system can be simplified when applying the discrete element simulation software to study the complex nonlinear motion process of the root-soil complex shearing the surrounding soil upward under the clamping lifting action of the green leafy vegetables orderly harvester.

The stems and leaves of green leafy vegetables are usually wrapped in 3–4 layers from outside to inside, with each layer consisting of four stems and leaves distributed around, and the layers are interleaved with each other to form a cruciferous shape. In this study, the modulus of elasticity, friction coefficient, and restitution coefficient of the root and stem of green leafy vegetables were measured experimentally, providing a crucial parameter basis for applying discrete element analysis to elucidate the orderly harvesting mechanism.

Studying the rheomechanical viscoelastic properties of green leafy vegetables can construct a mechanical plastic deformation damage equation and calculate the size of the damage. The creep experiment can effectively obtain the viscoelastic parameters of green leafy vegetables. Previous studies have shown that the orderly clamping device is an essential factor affecting the damage to green leafy vegetables to simulate the real force of green leafy vegetables in the clamping device. Thus, before conducting creep experiments, the spherical indenter and the sample placed above the base were wrapped around the conveyor belt to simulate the real mechanical action. Additionally, this study conducted compression creep experiments on whole green leafy vegetables and single leaves, comparing the creep of different samples under different pressures and providing a theoretical basis for high-quality, low-loss, orderly harvesting research, development, and device optimization design.

## 6. Conclusion

In this paper, the research on the physical, mechanical, and rheomechanical properties of green leafy vegetables was conducted systematically and comprehensively to address the problem of extensive mechanical damage during orderly harvesting of green leafy vegetables, the main contents of which are as follows:

1. Measurements were made on the roots and stems of several green leafy vegetables, and axial compression characteristic experiments were conducted using the TMS-Pro texture instrument. The elastic modulus E of the roots and stems of green leafy vegetables was 6.02 × 10^5^ Pa and 9.16 × 10^5^ Pa, respectively. The static friction coefficient, dynamic friction coefficient, and restitution coefficient between the root and the test material and between the stem and the test material were measured using the oblique method combined with high-speed photography. This provided data support for improving the discrete element modeling of green leafy vegetables and optimizing the orderly harvester.
2. Based on the rheomechanical properties of the viscoelastic body of green leafy vegetables, a complete and accurate mathematical constitutive equation characterizing its viscoelastic properties was established, and the main characteristic parameters affecting plastic deformation damage were analyzed. Using MATLAB data analysis software to fit the relationship deformation curve of the sample with time under constant pressure, the coefficient of determination R^2^ was higher than 0.996, indicating reasonable validity of the selected model. The total deformation of the sample under 60 s constant-pressure loading with different set loading forces was calculated, and the creep of different samples under different pressures was compared. This result provides a theoretical basis for the research and development of high-quality and low-loss orderly harvesting and the optimal design of the device.

## Supporting information

S1 File (ZIP)
