# Supplementary figures and images for: Experiment and analysis of physical, mechanical, and viscoelastic properties of the roots and stalks of green leafy vegetables
Source: PLoS One. 2024 Jul 2;19(7):e0305572. doi: 10.1371/journal.pone.0305572 (PMC11218987; doi:10.1371/journal.pone.0305572)

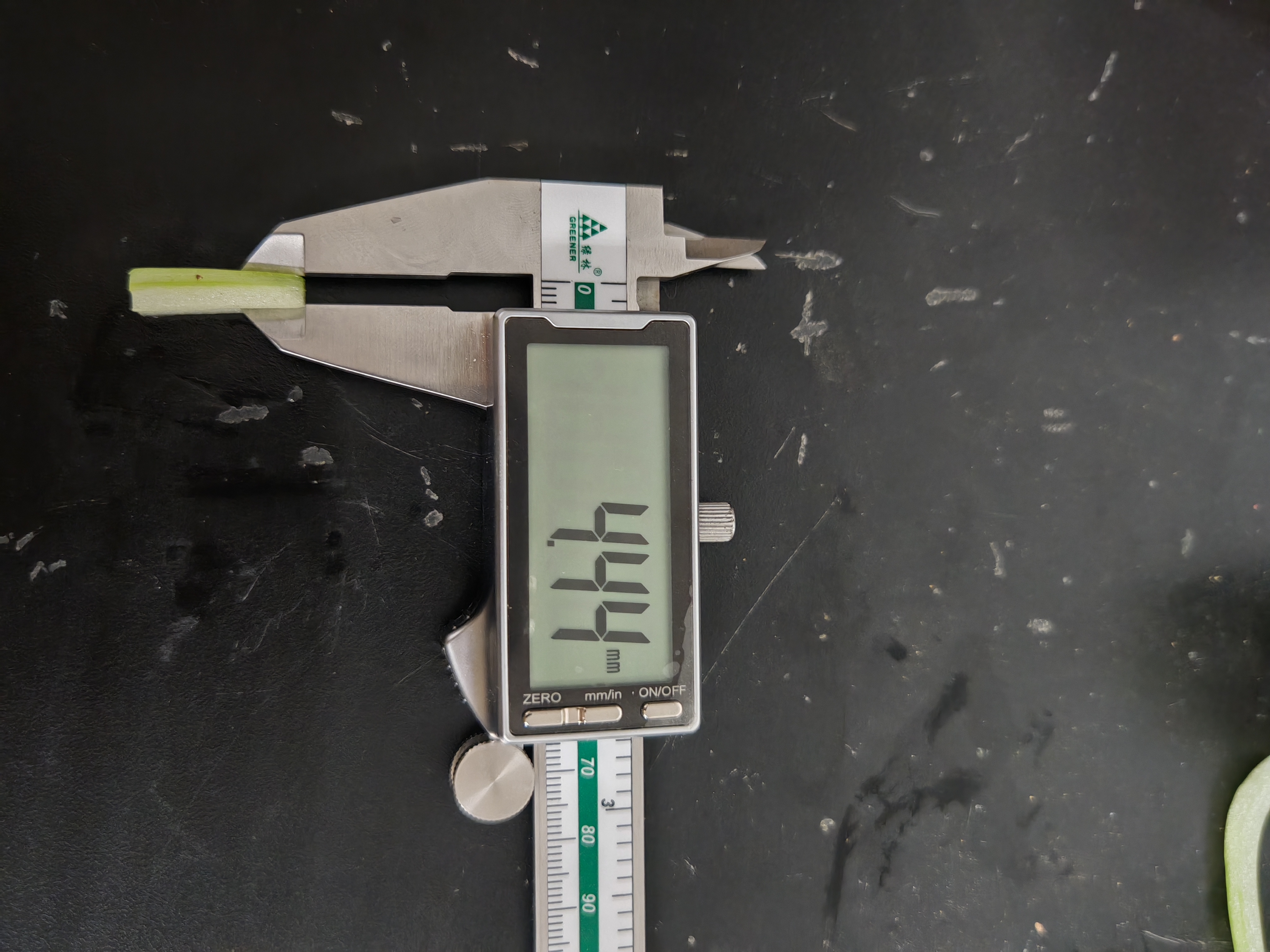

Supplement: S1 File — (ZIP) [file pone.0305572.s001.zip › Supplement the original image files/Fig 2 (1).jpg]

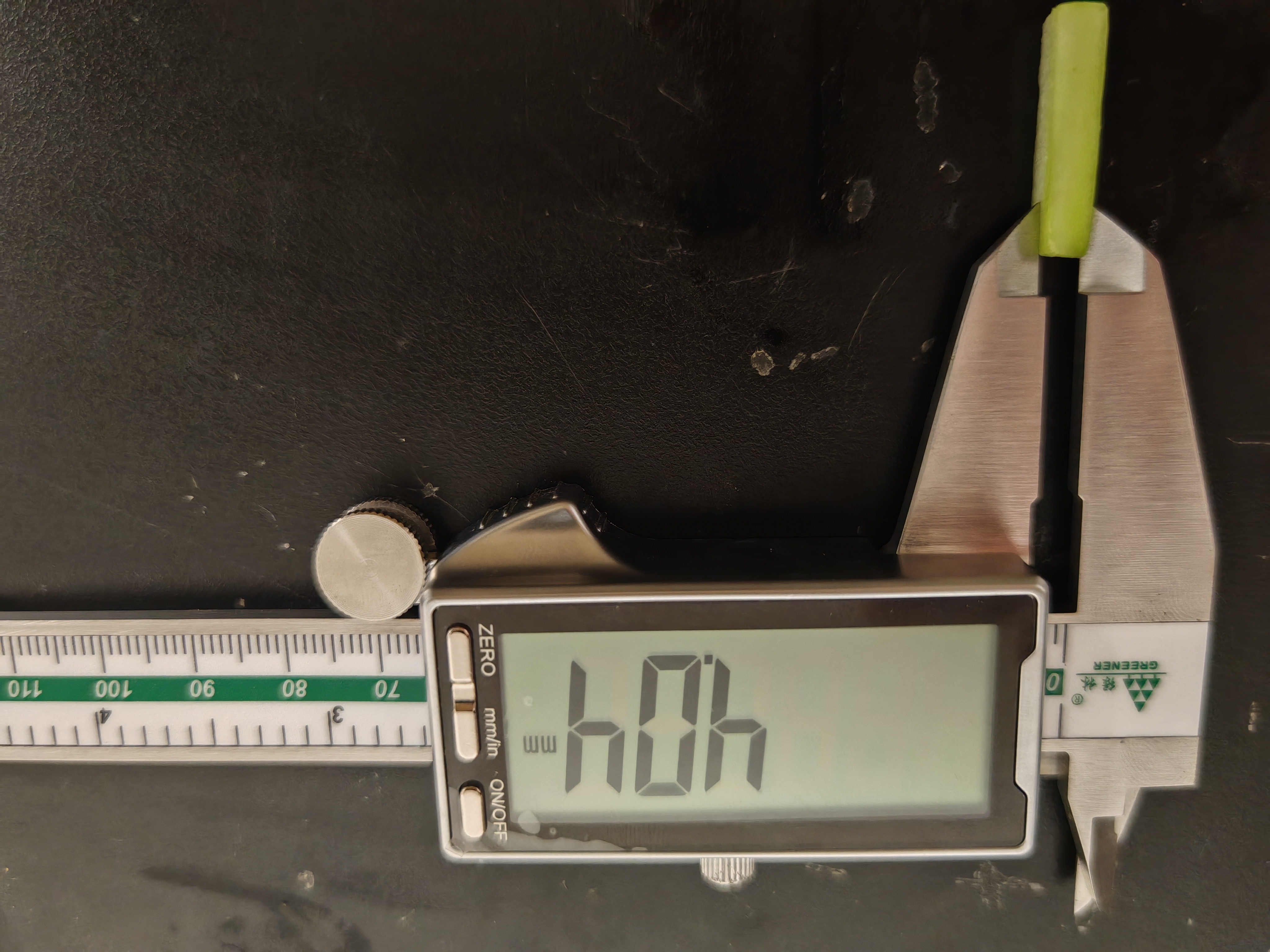

Supplement: S1 File — (ZIP) [file pone.0305572.s001.zip › Supplement the original image files/Fig 2 (2).jpg]

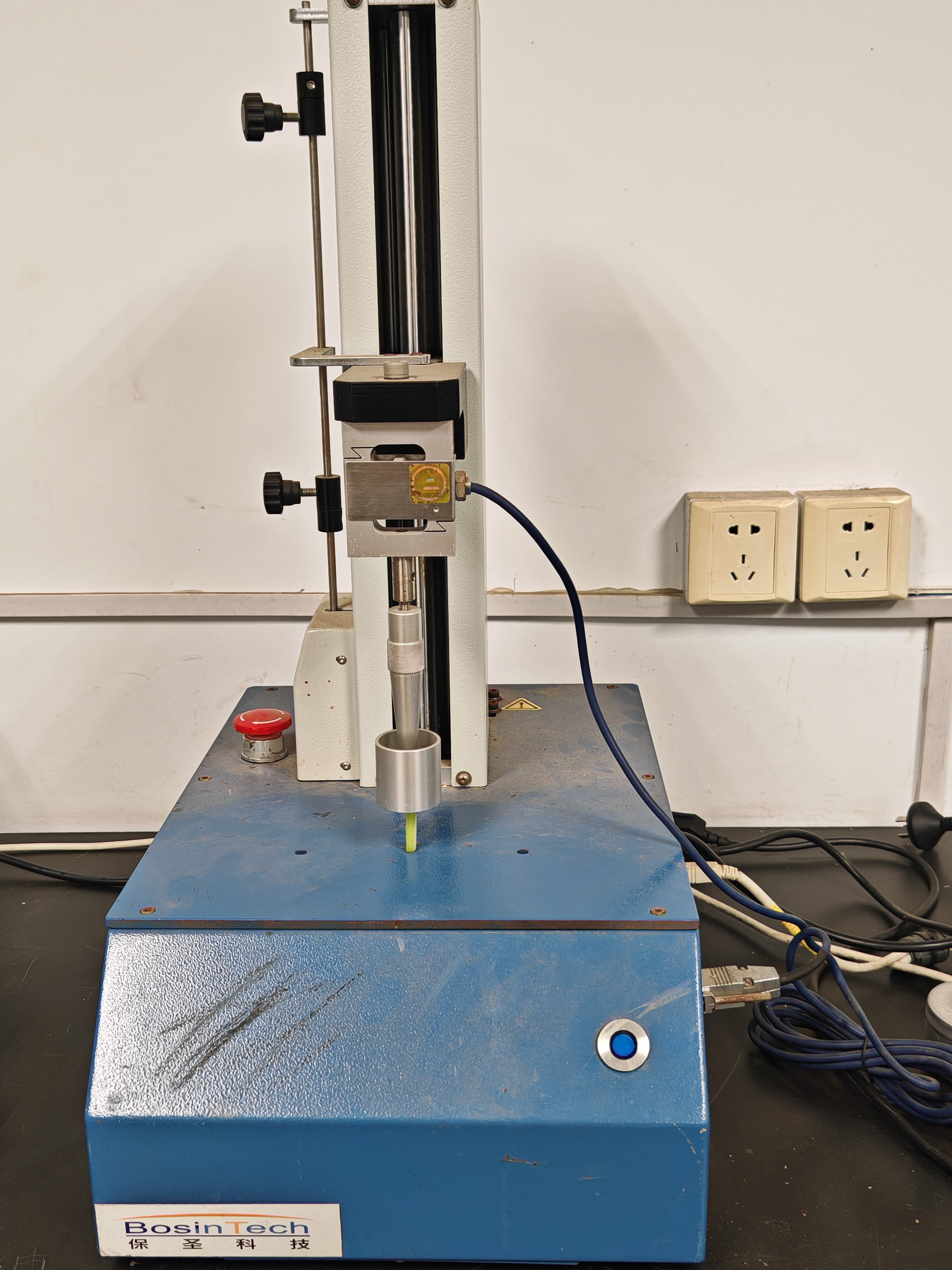

Supplement: S1 File — (ZIP) [file pone.0305572.s001.zip › Supplement the original image files/Fig 2 (3).jpg]

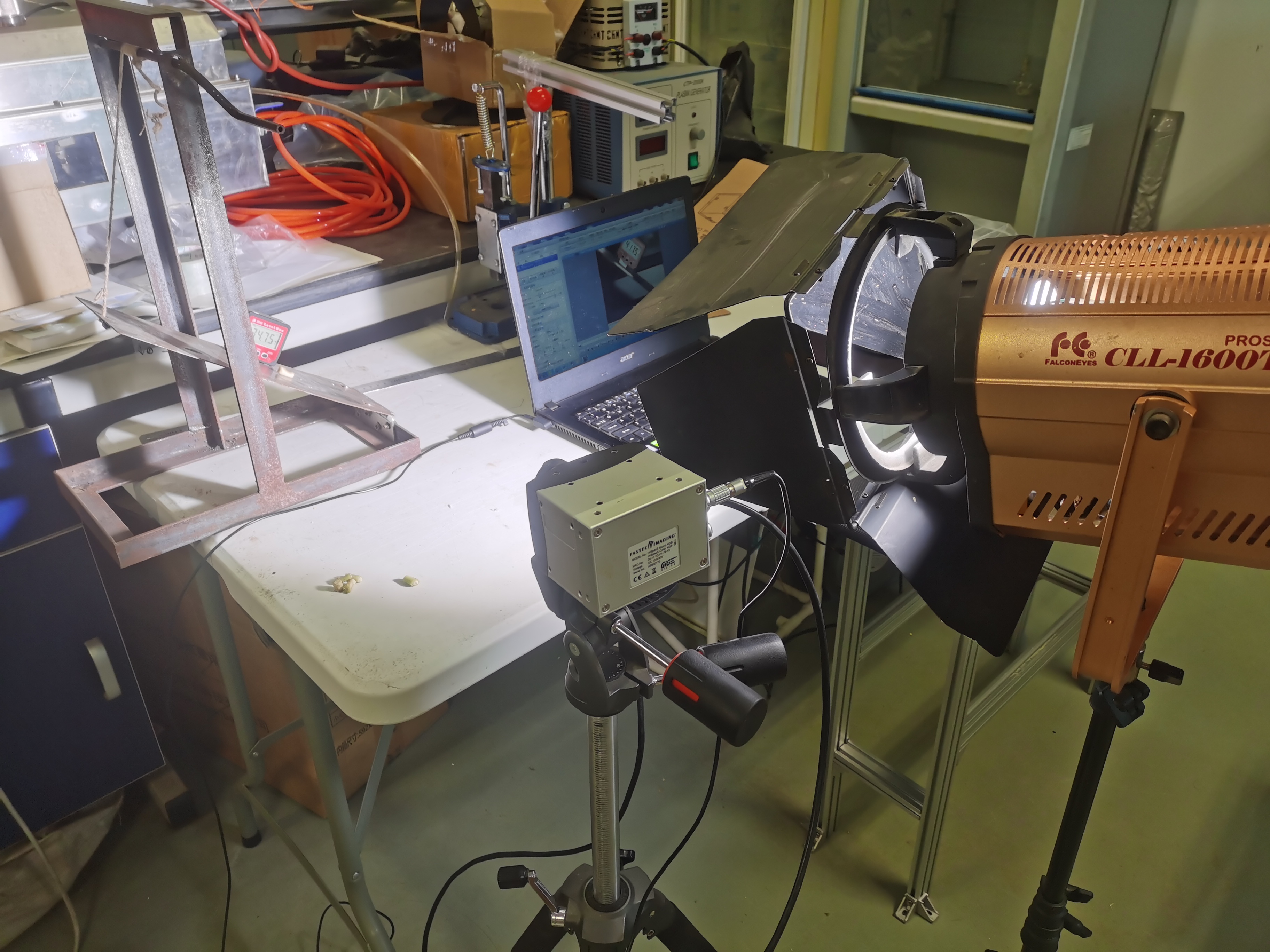

Supplement: S1 File — (ZIP) [file pone.0305572.s001.zip › Supplement the original image files/Fig 3.jpg]
